# Dr. Joseph H. Foster

**Published:** 1872-06

**Authors:** E. N. Harris

**Affiliations:** Cor'g. Sect'y.


### OBITUARY.

We regret to announce the death of one of the most estimable members of the Dental profession, Dr. Joseph H. Foster, of New York city. The following resolutions will show how highly Dr. Foster was regarded by his professional brethren:

At the monthly meeting of the American Academy of Dental Science, held in Boston, May 7th, 1872, the following resolutions were passed:

Whereas, it has pleased Divine Providence to remove from this world our brother and beloved friend, Joseph H. Foster, M. D., of New York, it is hereby

*Resolved*, That the American Academy of Dental Science receives the intelligence of the death of one of its most distinguished members with deep regret and heartfelt sorrow.

*Resolved*, That in the death of this estimable man, the Academy mourns an honored member, an able teacher, and one whose practice fully sustained the proudest and highest standard of requisitions known to the profession.

*Resolved*, That in the death of Dr. Foster, society has lost a useful and honorable citizen, and the social circle a faithful and beloved companion.

*Resolved*, That the proceedings of the Academy of this day, in honor of our late and lamented brother, be entered upon its records and communicated to the widow of the deceased, with the assurances of deep sympathy in her bereavement.

Also, that a copy of these resolutions be forwarded to the *American Journal of Dental Science* and to the *Dental Cosmos* for publication. E. N. Harris, D.D.S., Cor'g. Sect'y.

